# Investigating prevalence job stress and illness among hospital staff providing health tourism services (HSPHTS) in Iran

**DOI:** 10.1186/s12913-020-05761-x

**Published:** 2020-09-29

**Authors:** Farhad Hemmati, Fatemeh Dabbaghi, Ghahraman Mahmoudi

**Affiliations:** 1 Research, Hospital Administration Research Center, Sari Branch, Islamic Azad University, Sari, Iran; 2 Hospital Administration Research Center, Sari Branch, Islamic Azad University, Sari, Iran

**Keywords:** Illnesses, Job stress, Hospitals, Health tourism

## Abstract

**Background:**

Health tourism or treatment tourism is one of threatening fields that causes (added s) increase in prevalence of job stress and illnesses among hospital staff of providing health tourism services (HSPHT). The aim of this study is to determine the prevalence of job stress and illnesses among hospitals staff of providing health tourism services in touristic cities as Tehran and Shiraz in Iran.

**Methods:**

This cross-sectional, descriptive-analytical study was carried out among the staff of 10 hospitals providing health tourism services in cities of Tehran and Shiraz, Iran, in 2019. In these hospitals, 1250 staff were chosen by cluster sampling method from each job task as doctor, nurse, office worker, and paramedical and cleaner worker. Also, 1100 staff working in other general hospitals (non-HSPHTS) were selected as the control sample. Then, the demographic information and prevalence of job stress were gathered by Osipow job stress questionnaire and the illnesses were accumulated by self-reporting questionnaire. Finally, data were analyzed in SPSS 20 software. Chi-square and Pearson’s parametric tests were used in the study.

**Results:**

Prevalence of illnesses among HSPHTS was more than that in general hospitals (*P*_value_ < 0.05). The most prevalent illnesses were respiratory (11.08%), digestive (9.2%), and cutaneous (9.04%), respectively. Also, the prevalence of job stress among HSPHTS was more than that in general hospitals (*P*_value_ < 0.05). There was a significant relationship between prevalence of illnesses and job stress among the staff of hospitals and the increase in the number of visited tourists in the hospitals providing health tourism services.

**Conclusion:**

Results of the study showed that the prevalence of job stress (%33.76) and illnesses (%43.66) among the HSPHTS was respectively 2 and 2.6 times more than that among the staff of general hospitals. Thus, it is necessary to observe sanitary actions and considerations more seriously in these hospitals.

## Background

Health tourism or tourist treatment is one of the fields that can take a significant role in enhancing industrial tourism [1], in which Iran has recently improved [2, 3]. Development in this industry does not only affect the expression of national identity, but also causes development in economic dimensions via creating job opportunities, monetization, decreasing poverty, and improving social justice and welfare in the society [2, 4].

Although health tourism promotes health by seeking to improve the health of tourists, from a health perspective, it is one of the important dangers and threats to the health of the hospital staff providing health tourism services (HSPHTS). Therefore, it should be noted that along with the advantages of this field, the adverse effects include transmission and prevalence of contagious infectious diseases [5] and job stress [6] resulted from the high level of responsibility among the staff regarding the quality of services, satisfaction level, and higher health level of tourists [7–9].

There are many studies around the world about the transmission of infectious diseases among tourists through water and food, carriers, common diseases between humans and animals, sexual contact, blood, air, soil, and so on [10]. Studies have demonstrated that the prevalence rate of infectious illnesses among tourists in England is between 27 and 43% [10, 11]. Prevalence of illness was reported nearly 29% in the USA [12]. Also, these illnesses becomes about 39% when American military army travels [12, 13]. Raid et al. reported that the prevalence of illnesses among tourists between 10 and 30 years old (53%) was more than that among tourists over 40 years old (31%) [10]. However, despite the large number of foreign tourists and health tourism activities in the tourist country of Iran, there are no reports on the prevalence of illnesses and its effects on tourists and health tourism providers [7].

Furthermore, foreign studies have demonstrated that job stress is more prevalent among nurses and physicians working in the hospitals providing health tourism services, since they pay more attention to treating diseases and improving the health level of tourists [6, 8]. Also, making relationship of the hospital staff with different cultures of tourists and developing effective relationships in international communication have increased job stress among HSPHTS [7, 14, 15].

According to the statistics issued by Ministry of Health, 42 hospitals have obtained the license to provide health tourism services from Ministry of Health and Medical Education in the tourist cities of Iran including Mashhad, Tabriz, Esfahan, Shiraz, and Tehran; most of these hospitals are located in Tehran and Shiraz. Totally, about 18,000 medical staff, including physicians, nurses, office workers, paramedical and cleaner worker, etc. are working in these hospitals.

Although Iran is a tourist country and health tourism in this country in recent years has been improving [16], the transmission of tourists’ illnesses to the hospital staff is inevitable. Also, the quantity and quality of providing health services to foreign tourists are important and hospital staff feel more responsibility in terms of providing services, satisfying the patients, and promoting the health level among tourists. Therefore, the related job stress can significantly affect the hospital staff. So, this study aims to determine job stress and transmitted illnesses among HSPHTS in tourist cities of Iran, i.e. Tehran and Shiraz.

## Methods

This cross-sectional, descriptive-analytical study was carried out on the staff of 10 hospitals providing health tourism services in cities of Tehran and Shiraz, Iran, in 2019. Considering about 5000 staff including physicians, nurses, office workers, and paramedical and cleaner workers in these hospitals, 25% of the staff from each group was selected by cluster sampling method. Then, 125 staff from each of the 10 hospitals (5 hospitals in Tehran and 5 hospitals in Shiraz) were selected. So, the total staff samples of our study in the hospitals providing health tourism services was 1250 persons. Also, 1100 staff were selected as control in other general hospitals, so that they were the same as the experimental group in the number of physicians, nurses, office workers, and paramedical and cleaner workers. Cooperation call for research was sent to the administrators of hospitals providing health tourism services in Tehran and Shiraz to explain the purpose of the study and their contribution to the study. Five hospitals in Tehran and 5 hospitals in Shiraz declared their consent for cooperation. The researchers of this study were obliged to keep confidential the personal information of each of the staff of the hospitals providing health tourism services and the control participants. The participants were asked to leave the study whenever they felt any inconvenience in the study.

The demographic variables of the hospital staff were age, gender, type of job, education level, smoking status, alcohol use status, receiving health, and safety advice. The method of collecting illness-related data was self-reported. We asked a question from all the participants: “What kind of illnesses have you had in the last 6 months? [17]. Also, the standard Osipow questionnaire was used to evaluate the prevalence of job stress in these hospitals [18]. Osipow job stress questionnaire is scored on a five-point Likert scale from 1 to 5. The questionnaire was set in six dimensions of job stress and each dimension contains 10 questions.

The six dimensions of the questionnaire are work load, inefficiency of role, duality of role, range of role, responsibility, and physical environment, respectively. The scores for male and female were determined on the basis of the Osipow questionnaire and, accordingly, levels of job stress for every participant were determined. This scale is specified at four levels: Less than normal stress; Normal stress; Moderate stress; and Severe stress (Table 1). In this study, a person with moderate to severe stress was considered to have stress.

**Table 1:** Criteria for Scoring Job Stress Levels in Males and Females

Total stress	Male	Female
Less than normal	10–133	60–107
Normal	134–216	108–203
Moderate	217–258	204–251
Severe	259–300	251–300

The questionnaires were distributed among the HSPHTS and staff of general hospitals by trained experts. Two researchers were available to answer any questions from the participants.

Data analysis was performed in SPSS software (version 20). Frequency distribution, mean, standard deviation, and percentage were reported for every variable. The normality of every variable was then tested using Kolmogorov-Smirnov test with the error rate of ≥0.05. Chi-square and Pearson’s parametric tests were employed in the study. The correlation was also presented with the 95% confidence interval (CI) for the significant variables. For multiple comparison, Bonferroni correction was conducted by dividing the original α-value by the number of analyses on the dependent variable.

## Results

The mean age (SD) of the HSPHTS was 31.1 (± 1.3) years old for males and 27.4 (± 0.9) years old for females. These staff were working on two shifts, day (57%) and night (43%). The mean (SD) work experience for the males and females was respectively 7.5 (± 1.1) and 8.2 (± 1.9) years old. The mean age (SD) of general hospital staff as control samples was 30.3 (± 2.1) years old for the males and 29.3 (±1.7) years old for the females. These staff were working on two shifts, day (52%) and night (48%). The mean (SD) work experience for the males and females was 8.5 (±1.4) and 8.7 (±2.2) years, respectively. Other information of HSPHTS and non-HSPHTS is given in Table 2.

**Table 2:** Frequency of Socio-demographic Characteristics in Selected HSPHTS and non-HSPHTS

Variable	Non-HSPHTS		HSPHTS	
	N	%	N	%
**Gender**				
Male	539	49	563	45
Female	561	51	688	55
**Age**				
30 years old or younger	638	58	588	47
Above 30 years old	462	42	663	53
**Type of Job**				
Nursing	341	31	363	29
Cleaner worker	253	23	263	21
Office worker	154	14	163	13
Paramedical	220	20	325	26
Medical	132	12	138	11
**Level of Education**				
Primary education	341	31	150	12
Secondary education	330	30	425	34
College or university	429	39	675	54
**Smoking Behavior**				
Never smoking	737	67	1013	81
Ever smoking	363	33	238	19
**Alcohol Use**				
Never using	770	70	738	59
Ever using	330	30	513	41
**Health Advice**				
Yes	231	21	462	37
No	869	79	787	63
**Total**	1100	100	1250	100

The results of statistical tests showed a significant difference between the prevalence of illnesses in the HSPHTS and non-HSPHTS, as presented in Table 3.

**Table 3:** Comparing Prevalence of Illnesses among the HSPHTS and non-HSPHTS

Types of Illness	Non-HSPHTS		HSPHTS		*P*_value_
	N	(%)	N	(%)	
Respiratory	27	2.45	134	11.08	< 0.05
Dermal	26	1.36	113	9.04	< 0.05
Digestive	64	2.82	115	9.2	< 0.05
Neural	24	2.18	34	2.72	> 0.05
Kidney	25	2.30	50	3.65	> 0.05
Cardiovascular	12	1.08	54	4.33	< 0.05
Blood	18	0.64	25	2	> 0.05
Other	18	1.64	21	1.64	> 0.05
Total	178	16.47	546	43.66	< 0.05

Based on Table 3, the prevalence of respiratory, cutaneous, gastrointestinal, and heart illnesses among HSPHTS was higher than that among the staff of general hospitals (*P*_value_ < 0.05). The highest prevalence of illnesses was related to respiratory (11.08%), gastrointestinal (9.2%), and cutaneous (9.04%) among the HSPHTS and non-HSPHTS. The least prevalence of illnesses was observed in neurological, renal, blood, and other diseases. Overall, the total prevalence of illnesses among the HSPHTS was 43.66%, which was about 2.6 times of the staff of general hospitals staff (16.47%). The results of the statistical tests showed, in general, there was a significant difference between the prevalence of job stress among the HSPHTS and the staff of the general hospital (*P*_value_ < 0.05), as presented in Table 4.

**Table 4:** Comparing Prevalence of Job Stress among the HSPHTS and non-HSPHTS

Total stress	Non-HSPHTS		HSPHTS		*P*_value_
	N	(%)	N	(%)	
Less than normal	349	31.7	217	17.36	< 0.05
Normal	558	50.7	504	40.32	< 0.05
Moderate	106	9.6	228	18.24	> 0.05
Severe	87	7.9	194	15.52	< 0.05
Stressed persons	193	17.5	422	33.76	< 0.05
Normal persons	907	82.5	828	66.24	> 0.05
*P*_value_	< 0.05				

Based on Table 4, the prevalence of job stress among HSPHTS was higher than that among the non-HSPHTS (*P*_value_ < 0.05). Overall, the prevalence of job stress (moderate and severe) among the HSPHTS (33.76%) was about twice of the non-HSPHTS (17.5%).

**Fig. 1:**
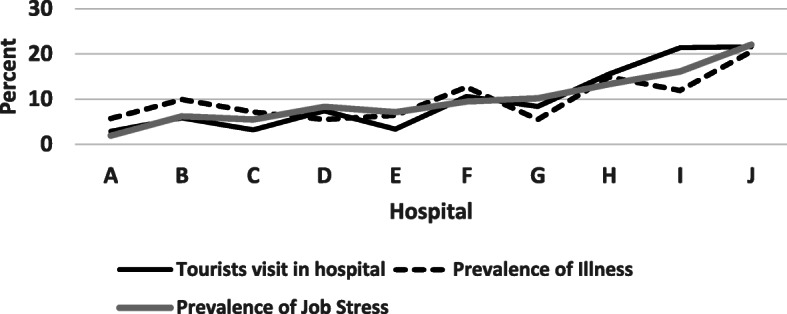
Relationship between prevalence of illnesses and occupational stress among the HSPHTS and tourists visited rate for getting health tourism services

The results showed that the prevalence of illnesses and job stress among HSPHTS was affected by the rate of tourists visited for receiving health tourism services, as shown in Fig. 1.

About 36,000 tourists were registered for receiving health tourism services in the studied hospitals in 2019. The contribution of each of the studied hospitals to health tourism services was different, so that 55% of the health tourism services was related to three hospitals, while about 50% of job stress and illnesses of the hospital staff were related to these three hospitals.

The results of the statistical analysis showed a significant relationship between the number of visited tourists for health tourism services in hospitals and the prevalence of illnesses and job stress among the HSPHTS (*P*_value_ < 0.05). Figure 1 shows that increase in the number of visited tourists for health tourism services led to high prevalence of illnesses and job stress among the hospital staff (*P*_value_ < 0.05).

Table 5 shows the relationship of some of the socio-demographic characteristics associated with the prevalence of illnesses and job stress among the HSPHTS.

**Table 5:** Relationship between Socio-demographic Variables and Illnesses and Job Stress among the HSPHTS

Variable	Illness	***P***_value_	Stress	***P***_value_
	N (%)		N (%)	
**Gender**				
Male	349 (64)	< 0.05	300 (71)	< 0.05
Female	197 (36)		122 (29)	
**Age**				
30 years old or younger	267 (49)	> 0.05	224 (53)	< 0.05
Above 30 years old	279 (51)		198 (47)	
**Type of Job**				
Nursing	158 (29)	< 0.05	118 (28)	< 0.05
Cleaner worker	169 (31)		84 (20)	
Office worker	66 (12)		80 (19)	
Paramedical	82 (15)		68 (16)	
Medical	71 (13)		72 (17)	
**Academic Year**				
Primary education	207 (38)	< 0.05	186 (44)	< 0.05
Secondary education	218 (40)		139 (33)	
College or university	120 (22)		97 (23)	
**Smoking Behavior & Alcohol Use**				
Never	180 (33)	< 0.05	165 (39)	< 0.05
Ever	366 (67)		257 (61)	
**Health Advice**				
Yes	158 (29)	< 0.05	114 (27)	< 0.05
No	333 (61)		308 (73)	

The results of statistical tests in Table 5 showed that the prevalence of illnesses and job stress among the males was higher than that among the females in the HSPHTS (*P*_value_ < 0.05). There was no significant relationship between the prevalence of illnesses and age (*P*_value_ > 0.05). However, there was a significant relationship between the prevalence of job stress and age among HSPHTS. Also, job stress was higher among the personnel younger than 30 years old (*P*_value_ < 0.05). The results showed that the prevalence of illnesses and job stress was significantly different in different jobs and was higher among nurses and cleaner workers (*P*_value_ < 0.05). There was a significant difference between education level and prevalence of job stress and illnesses and the prevalence of job stress and illnesses was related to the staff with lower level of education (*P*_value_ < 0.05). The prevalence of job stress and illnesses was higher among the personnel who were smoking and consuming alcohol (*P*_value_ < 0.05). The results showed that health advice had a positive effect on decreasing the prevalence of job stress and illnesses among the HSPHTS (*P*_value_ < 0.05).

## Discussion

The results of this study represented that the prevalence of job stress (33.76%) and illnesses (43.66%) among the HSPHTS was about 2 and 2.6 times higher than that of the staff in general hospitals (control). The most prevalent diseases were respiratory (11.08%), gastrointestinal (9.2%), and cutaneous (9.04%), respectively. Also, the prevalence of job stress and illnesses among the nurses and cleaner workers, males, smokers and alcohol users, and those with low level of education were higher than that among the other staff.

In the 2017 study by Vong et al. in Macau, the most important result was that the stress of nurses serving tourists was higher than other nurses. The results of our study are the same as their results [9]. Probably, job stress is likely to be higher among the personnel who are in direct relation with health tourism, since they pay more attention to illness treatment, promote tourists’ health level, and take effective measures in international communications and the country’s economy.

In many studies, the transmission of illnesses has been discussed by tourists. Tourists are considered to be carriers of infectious diseases and transfer the diseases to the personnel providing services to them, including HSPHTS [5, 11]; this issue should be considered hygienically [6, 14]. Also, the important results of our study determined that tourists have spread diseases among the HSPHTS, especially nurses and cleaner workers in the studied hospitals, which can be a serious threat to the spread of other dangerous illnesses. The reason for the prevalence of these diseases might be that the studied personnel were more exposed to sick tourists in order to provide better services and attract tourist satisfaction [7].

The results of our study indicated that the prevalence of job stress and illnesses was higher among males, smokers, and alcohol consumers. The reason can be due to paying less attention to health laws. Also, these staff show unpredictable behaviors due to the use of these substances. The reason for the high job stress among these staff may be that they think they are not doing their task properly. On the other hand, other results show that the prevalence of job stress and illnesses among low-educated staff is higher, which is probably due to the lack of training and having essential skills needed to deal with visited tourists as well as lack of control on the medical and hygienic services.

There were some limitations in this study that should be taken into consideration when interpreting the results. The cross-sectional design of the study and self-reporting of the collected data may not allow for making actual causative conclusions. Furthermore, since the current research was conducted among of the HSPHTS with conservative data, bias in the collected data may have affected the results. In this study, age can be an intervening factor in proving the role of work experience in causing occupational stress and diseases, which could be probably due to the lack of proper selection of samples in different age groups.

## Limitation

Due to the fact that the first research in this field has been done in our country, so there are some limitations.

One of the limitations of this study was that it was only performed in Tehran and Shiraz. Limiting the results to these two cities might lead to selection bias. It is recommended for future studies to investigate other towns that have hospitals providing health tourist services. Another limitation was the lack of cooperation on the staff side and inadequate completion of the questionnaires by them. For overcoming this problem, expert researchers were selected from the beginning, and all the required training was provided for them by the research team to decrease the information bias. Yet, another limitation was that this study was cross-sectional; thus, it was not possible to assess the temporal priority while investigating the relationship of illnesses and job stress, on the one hand, and smoking and alcohol consumption, on the other hand. Therefore, it is recommended to perform a case-control study in the future.

## Conclusion

Results of this study showed that the prevalence of job stress and illnesses among the HSPHTS was about 2 and 2.6 times higher than that of the general hospital staff (control), respectively. Due to the relatively high prevalence of occupational stress and illnesses among the HSPHTS, it is necessary to pursue health actions and interventions more seriously in these hospitals.

## Data Availability

The datasets generated and/or analyzed during the current study are not publicly available due to joint research and development with the company, but are available from the corresponding author on reasonable request.

## Abbreviation

HSPHTS: Hospital staff providing health tourism services
